# The fin-to-limb transition as the re-organization of a Turing pattern

**DOI:** 10.1038/ncomms11582

**Published:** 2016-05-23

**Authors:** Koh Onimaru, Luciano Marcon, Marco Musy, Mikiko Tanaka, James Sharpe

**Affiliations:** 1 Systems Biology Program, Centre for Genomic Regulation (CRG), The Barcelona Institute of Science and Technology, Dr Aiguader 88, Barcelona 08003, Spain; 2 Universitat Pompeu Fabra (UPF), Barcelona, Spain; 3 Graduate School of Bioscience and Biotechnology, Tokyo Institute of Technology, B-17, 4259 Nagatsuta-cho, Midori-ku, Yokohama 226-8501, Japan; 4 Institució Catalana de Recerca i Estudis Avançats (ICREA), Passeig Lluís Companys 23, Barcelona 08010, Spain

## Abstract

A Turing mechanism implemented by BMP, SOX9 and WNT has been proposed to control mouse digit patterning. However, its generality and contribution to the morphological diversity of fins and limbs has not been explored. Here we provide evidence that the skeletal patterning of the catshark *Scyliorhinus canicula* pectoral fin is likely driven by a deeply conserved Bmp–Sox9–Wnt Turing network. In catshark fins, the distal nodular elements arise from a periodic spot pattern of *Sox9* expression, in contrast to the stripe pattern in mouse digit patterning. However, our computer model shows that the Bmp–Sox9–Wnt network with altered spatial modulation can explain the *Sox9* expression in catshark fins. Finally, experimental perturbation of Bmp or Wnt signalling in catshark embryos produces skeletal alterations which match *in silico* predictions. Together, our results suggest that the broad morphological diversity of the distal fin and limb elements arose from the spatial re-organization of a deeply conserved Turing mechanism.

## Abstract (web summary)

Mouse digit patterning is controlled by a Turing network of Bmp, Sox9, and Wnt. Here, Onimaru *et al*. show that fin patterning in the catshark, *Scyliorhinus canicula*, is controlled by the same network with a different spatial organization; thus, the Turing network is deeply conserved in limb development.

## Graphical abstract

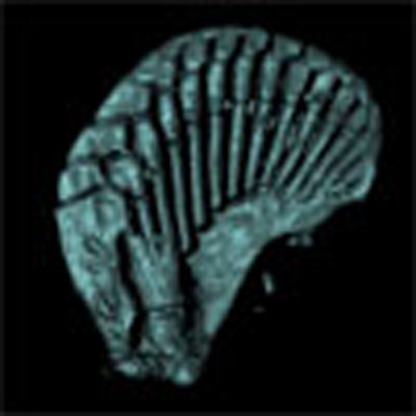

Despite the remarkable diversity of animal shapes, the repertoire of genes used for morphogenesis is unexpectedly conserved between species—an observation termed deep homology^1^. How similar sets of genes can govern the generation of diverse morphologies during evolution, however, is still not well understood. The fin-to-limb transformation is a paradigmatic example of morphological evolution^1^. The distal skeletal patterns of vertebrate limbs and fins have changed multiple times during evolution (Fig. 1a), and their homologous relationships have been controversial^2^,^3^,^4^,^5^,^6^,^7^. According to comparative anatomy, digits are regarded as a novel structure of tetrapod limbs, and do not trace back to non-tetrapod sarcopterygian fins^2^,^8^ (for example, *Sauripterus*^9^ and *Panderichthys*^7^). Yet despite the clear skeletal differences, recent molecular studies show unexpected similarities between the distal fins and limbs at the genetic level. For example, the digit-specific regulatory sequence of the murine *Hoxa* and *d* genes has recently been found in the genomes of the skate and the spotted gar, where they also drive similar expression in the distal fin/limb bud^6^,^10^. Thus a deep question remains: how can the skeletal arrangement change so markedly, when the well-known patterning genes do not?

Recent studies^11^,^12^ have provided strong evidence that digit patterning in the mouse limb is driven by a Turing mechanism, which has long been suggested theoretically^13^,^14^,^15^,^16^. Specifically, it has been proposed that regulatory interactions between BMP, SOX9 and WNT form a Turing network that creates a periodic molecular pre-pattern specifying the positions of the digits (the BSW model)^12^. A Turing model can generate different types of patterns, such as spots and stripes with only slight changes in parameter values. Therefore, we explored whether the BSW model could explain the marked changes in the distal skeletal arrangement of fins and limbs.

In this study, we focus on the pectoral fin development of the catshark, *Scyliorhinus canicula* for two reasons: (a) its fin skeletal elements are formed by individual condensations^17^, which are similar to the condensation process of tetrapod limbs; and (b) its genome is less derived than that of teleost genomes^18^. We show that spots of *Sox9* expression underlie the distal elements of *S. canicula* pectoral fin buds. In addition, by building a computer model, we demonstrate that such spot-like *Sox9* expression can be explained by the BSW model with slight modification of its parameters. Together, our results suggest that the broad morphological diversity of the distal fin and limb elements arose from the spatial re-organization of a deeply conserved Turing mechanism.

## Results

### The first periodic expression of *Sox9* is a distal row of spots

**Figure 1:**
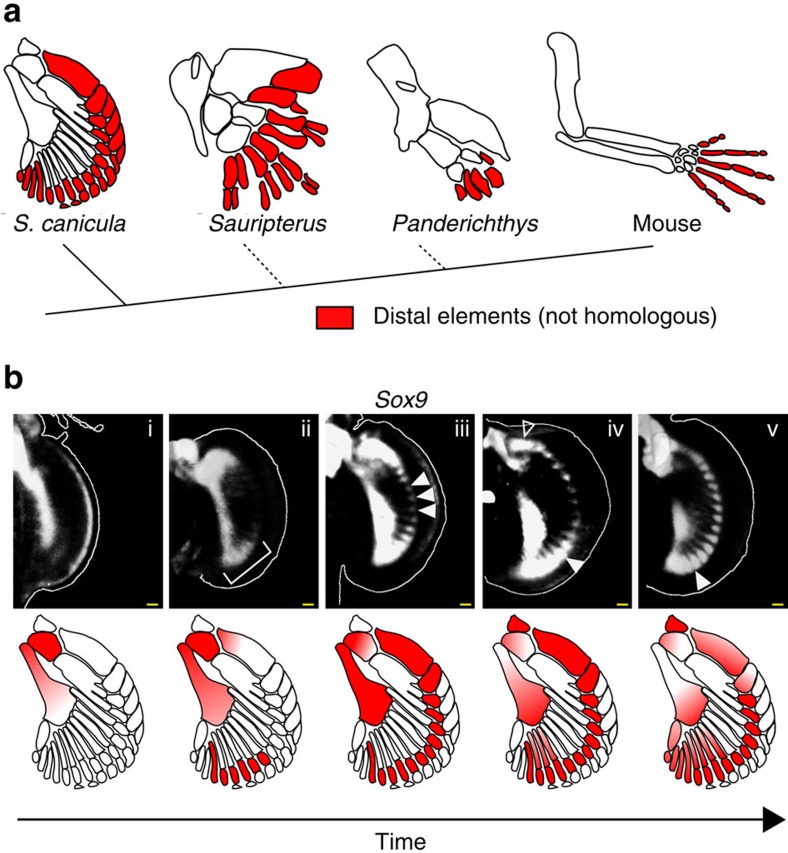
Time course of *Sox9* expression in *S. canicula* pectoral fin buds reveals the distal spot pattern. (**a**) Skeletal patterns of *S. canicula* pectoral fin, fossil fins^7^,^9^ and mouse limb. Red colours, distal elements. (**b**) Upper row shows OPT scans of *Sox9* expression in *S. canicula* pectoral fin buds at stages 29–30. (Dorsal view; anterior is to the top, and distal is to the right). The corresponding lower panels indicate (in red) which part of the future skeleton is represented by the Sox9 pattern above. Scale bars, 100 μm.

To understand the dynamics of skeletal patterning in *S. canicula* pectoral fin buds, we first examined a time course of *Sox9* expression using optical projection tomography (OPT)^19^. Our data revealed that the formation of distal nodular radials can indeed be captured in detail by *Sox9* expression, which is initiated as a single row of spots along the anterior–posterior axis (Fig. 1b). In more detail: at early stages *Sox9* starts to be expressed in the basal elements (Fig. 1b–i), and in the posterior-distal region (bracket in Fig. 1b–ii). Subsequently, a curved row of spots develop (arrowheads in Fig. 1b–iii), which is initially more continuous in the posterior region, but gradually also breaks up into spots (arrowheads in Fig. 1b–iv and v). These *Sox9* spots can be identified as the second row of distal nodular elements of the final skeleton (see Supplementary Fig. 1a–c for the later stages and the detailed annotation). A previous study reported a roughly similar expression pattern for *Sox8* (ref. 17), but distinct spots were hard to discern as a three-dimensional imaging technique (such as OPT) had not been used. Our data therefore reveals that the first stage of radial patterning is a dynamic specification of a spot-like pattern, in contrast to the stripy *Sox9* pattern of the mouse limb bud. We thus focused our study on control of this spot pattern (rather than subsequent expression of *Sox9* proximally or distally) for two reasons: firstly, we are interested in the initial symmetry-breaking process responsible for the overall radial arrangement, and secondly because previous studies suggest that the mechanism of patterning the distal periodic elements shows molecular differences from those controlling more proximal elements^12^,^20^.

### Out-of-phase patterns of Bmp and Wnt expression with *Sox9*

**Figure 2:**
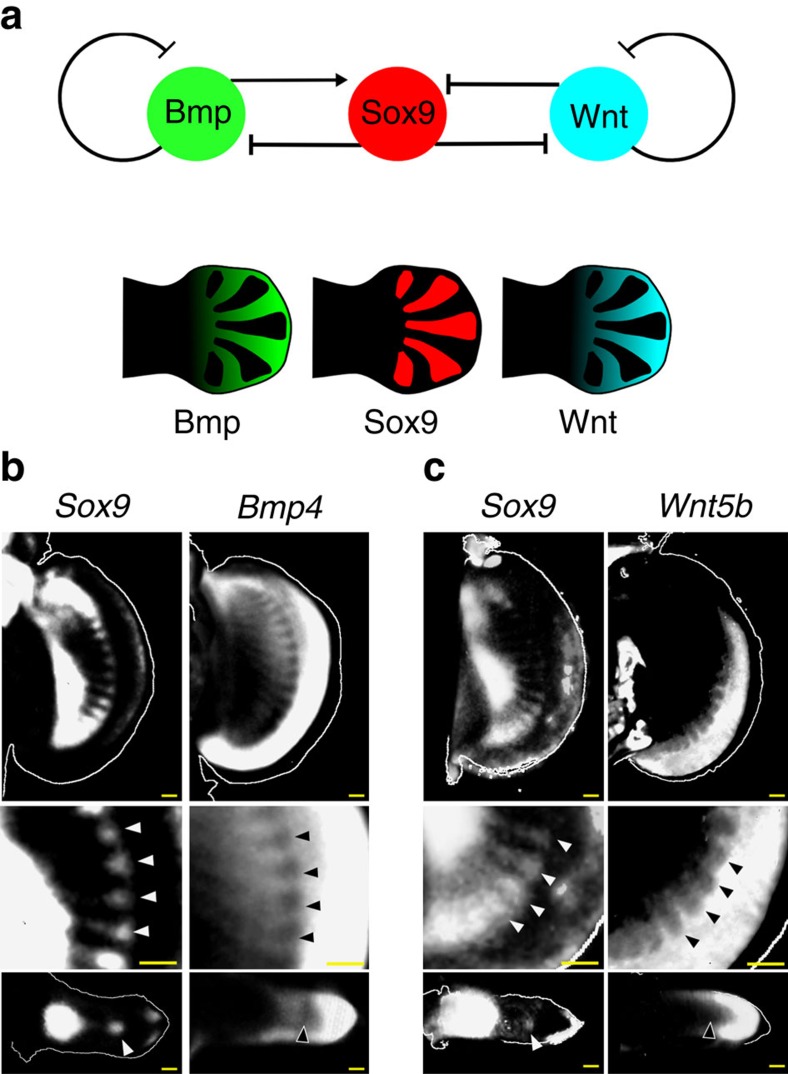
Out-of-phase expression patterns of *Bmp* and *Wnt* with *Sox9*. (**a**) The generic Turing network in mouse digit patterning, and distribution of Bmp expression (green), Sox9 expression (red) and Wnt signalling activity (blue). (**b**) Top row, OPT scans of gene expression patterns in the left and right pectoral fin buds of the same *S. canicula* embryo (stage 30). The *Sox9* image has been horizontally flipped to aid comparison of the two expression patterns. The middle panels (below) show magnified views of the top panels, highlighting with arrowheads the spots of *Sox9* expression and the corresponding gaps in the *Bmp4* expression. The lower panels show perpendicular virtual sections of the same two fin buds (‘transverse' sections) revealing that both the *Sox9* spot and the *Bmp4* gap, are in the centre of the bud, matching the situation in the mouse. Dorsal is to the top. (**c**) The same analysis as (**b**) for the *Wnt5b* gene. Scale bars, 100 μm.

If the patterning of the *S. canicula* pectoral fin was controlled by a Turing system similar to that controlling mouse digit patterning^12^, Bmp and Wnt might be expressed or active in a pattern out-of-phase with *Sox9* (Fig. 2a). We thus examined expression of Bmp- and Wnt-related genes in the *S. canicula* pectoral fin buds. Firstly, we found that *Bmp2* was expressed only in the distal fin edges, whereas in mice it displays the strongest out-of-phase pattern with *Sox9* (ref. 12) (Supplementary Fig. 2a). Instead, *Bmp4* was expressed in the fin mesenchyme and indeed has a pattern complementary to *Sox9* (Fig. 2b and Supplementary Fig. 2d; white and black arrowheads indicating a row of expression gaps where *Sox9* has a row of spots). Next, we examined genes related to Wnt signalling (Supplementary Fig. 2b,c,e). *Wnt5b* had a pattern out-of-phase of *Sox9* (Fig. 2c; Supplementary Fig. 2e), and a Wnt target gene *Lef1* (ref. 21) also showed a shallow pattern complementary to *Sox9* (Supplementary Fig. 2c). We also confirmed the complementary expressions by staining adjacent serial sections with either *Sox9/Bmp4* or *Sox9*/*Wnt5b* (Supplementary Fig. 2f). Thus, although several differences were found, the overall relationship between Bmp, Sox9 and Wnt is conserved from fish to mammals.

### A dynamical model of *S. canicula* fin development

**Figure 3:**
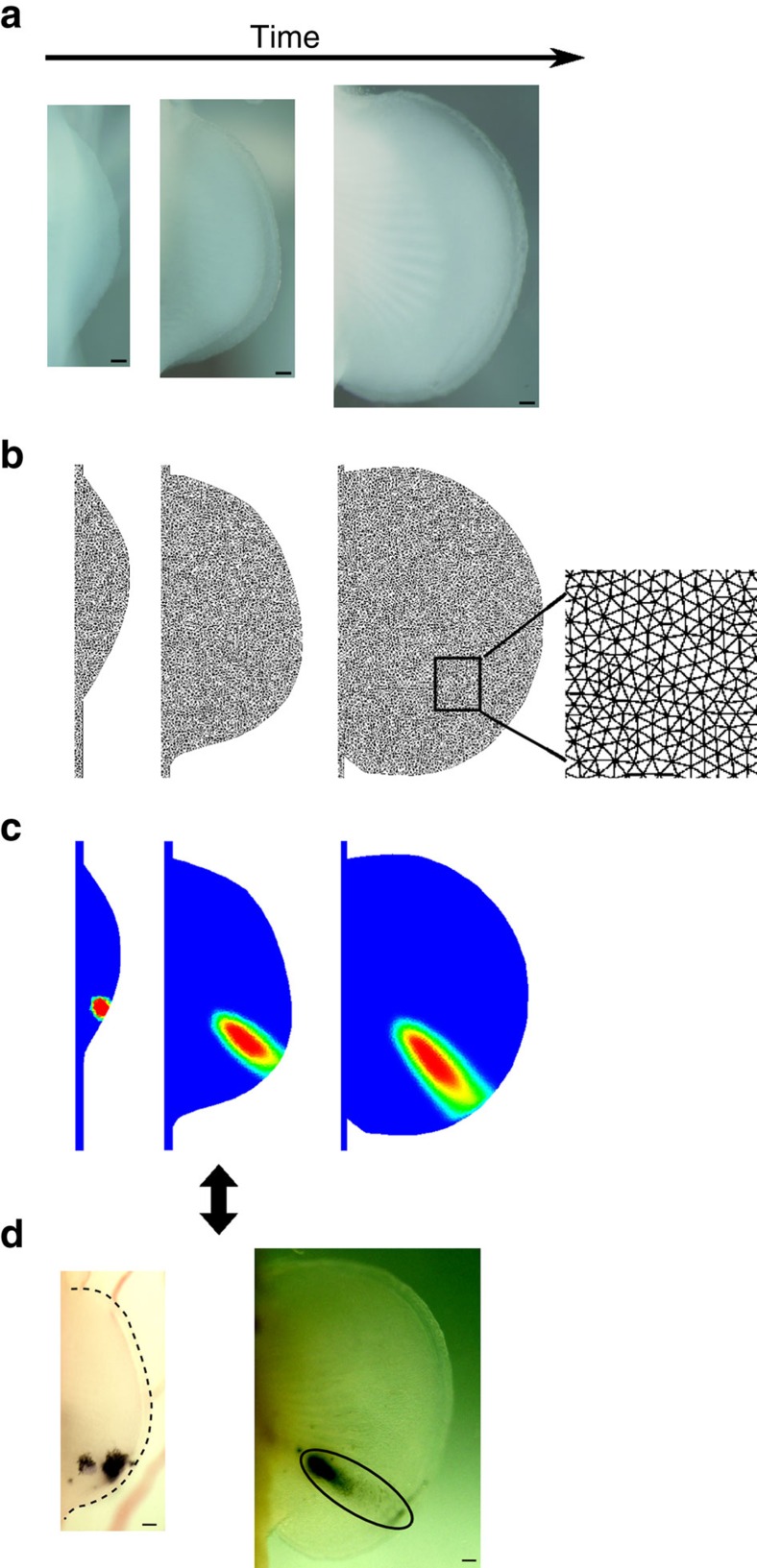
Construction of a fin growth model. (**a**) Dorsal views of *S. canicula* pectoral fin buds from stages 26 to 32. Anterior is to the top. Outlines of these fin buds are used for the fin growth model. (**b**) The fin growth model from stage 26 to stage 31. The spatial domain at each time point is discretized by a fine triangular mesh (see magnified view in the oldest fin shape). (**c**) An example of virtual fate map analysis. A small group of triangular elements are labelled with a virtual dye (concentration equal to one) at the earliest stage and the fate of the dye is simulated using the sequence of deformations and interpolations of the growth model. (The probability distribution of ink concentration is shown from red (high) to blue (low)). (**d**) Hypothetical growth maps are then compared to real fate map analysis with Indian ink. Scale bars, 100 μm.

To confirm if a BSW Turing network could reproduce the early spot pattern of the *S. canicula* fin, we built a realistic computational model using a similar approach to our previous mouse limb model^22^. In particular, we obtained a time course of pectoral fin morphologies, created a series of two-dimensional (2D) triangular meshes, and calculated hypothetical tissue trajectories which represent possible growth maps (Fig. 3; Supplementary Fig. 3). A crucial step was to determine how to align the chronological series of fin shapes (Supplementary Fig. 4a). To constrain this configuration, we required real fate map data, and thus despite the very slow growth of *S. canicula* fins we performed carbon-particle-based fate mapping^23^ (which required a minimum of 30 days to observe sufficient displacement of labelled tissue). By comparing real tissue displacements with *in silico* predictions, we could derive a realistic computational growth map, in which the posterior part of pectoral fin bud expanded more than the anterior part (Supplementary Fig. 4b–e). Interestingly, the asymmetry in growth observed along anterior–posterior axis is consistent with the fate maps observed in chick limb buds^24^,^25^.

### *In silico* modelling of the spot-type Sox9 expressions

**Figure 4:**
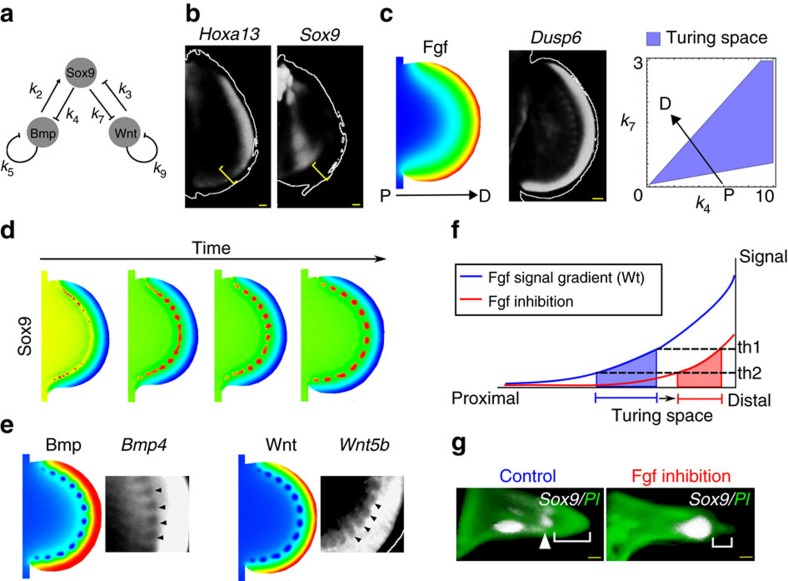
The *in silico* Turing model can reproduce the spot pattern of *Sox9* expression. (**a**) The generic Turing network model. (**b**) *Hoxa13* and *Sox9* expression in the right and left pectoral fin buds of the same embryo at stage 30. Yellow bracket: the distance of *Sox9* expression from the fin edge, indicating non-significant overlap with *Hoxa13*. (**c**) The simulated Fgf gradient (left) corresponds well to the experimental expression pattern of an Fgf target gene, *Dusp6* (centre). Right: parameter space of *k*_4_ and *k*_7_ (see **a**) indicates which combinations of parameter values lead to a Turing pattern (blue region). The arrow indicates how *k*_4_ and *k*_7_ change along the Fgf gradient, passing from low proximal Fgf (P) to high distal Fgf (D). This ensures that the spot pattern of Sox9 occurs only within a certain distance range from the AER. (**d**) The time course of a simulation result of the BSW model on the fin growth model. Red to blue colours indicate high to low concentrations of Sox9 (*S*). (**e**) The distribution of Bmp (*B*; left) and Wnt (*W*; right) concentration at the final time of the simulation matches the real expression patterns. In particular, shallow gaps of expression (black arrowheads) occur within a steep PD gradient. (**f**) Illustration of how Fgf inhibition changes the position of Turing space. Space is represented on the *x* axis, and the strength of Fgf signal on the *y* axis. A Turing pattern forms between the two threshold activity levels th1 and th2. When the normal Fgf signalling gradient (blue) is repressed (red), the position of the Turing pattern (shaded regions) shifts distally. (**g**) Virtual sections of *S. canicula* fin buds show *Sox9* expression (white) in Control (DMSO, *n*=8/8) or Fgf inhibited (SU5402 *n*=7/12) experiments (stage 30). In the latter case the distance from distal *Sox9* expression to the edge of the fin bud (square brackets) is reduced. Dorsal is up, distal is right. Green, nuclear staining with propidium iodide (PI). Arrowhead, the position of distal *Sox9* expression. Scale bars, 100 μm.

Because of the overall conservation of the distribution of Sox9, Bmp and Wnt, we next explored whether the *S. canicula* distal fin elements could also be specified by the BSW model^12^, which is expressed by the following partial differential equations:


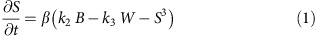



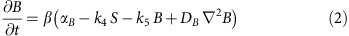



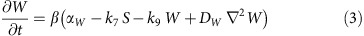


where *S*, *B* and *W* are abstract variables representing the amounts of Sox9, Bmp and Wnt expression, respectively, *k*_2_ to *k*_9_ are kinetic parameters (Fig. 4a), *D*_*B*_ and *D*_*W*_ are diffusion constants of *B* and *W*, respectively, *α*_*B*_ and *α*_*W*_ are constant production terms of *B* and *W*, respectively, and *β* is a global coefficient that controls the speed of pattern appearance. This system is composed of one non-diffusive molecule, Sox9 (*S*) and two diffusive molecules, Bmp (*B*) and Wnt (*W*). Our numerical simulations revealed that the model formed spots instead of stripes when Wnt production was significantly higher than Bmp production (Supplementary Fig. 5a). When we simulated with this condition in the fin growth model, a uniform distribution of spots emerged that had no resemblance to the real *Sox9* expression patterns (Supplementary Fig. 5a).

Previous work in the mouse has shown that distal *Hox* genes and fibroblast growth factor (FGF) signalling provide spatial modulation of the Turing network to sculpt the *Sox9* pattern into the normal digit arrangement^12^. We thus hypothesized that these molecules could play in a role in shaping the *Sox9* expression into a curved row of spots at a certain distance from the distal fin edge. In the mouse model, *Hoxd13* restricts the domain where the Turing instability occurs. Because in *S. canicula*, *Hoxa13* instead of *Hoxd13* is significantly expressed in the distal fin buds^17^,^26^, we first examined *Hoxa13* expression with OPT, but found that the *Hoxa13* expression domain did not overlap significantly with the distal expression of *Sox9* (Fig. 4b). Hence, we ruled out a similar role of *Hoxa13* in *S. canicula* fin bud.

We therefore asked whether Fgf alone could control the Sox9 spot pattern. We simulated an Fgf gradient by assuming that the ligand is produced at the distal fin edge and diffused towards the proximal part (Fig. 4c). The shape of the resulting gradient was roughly similar to the expression domain of an Fgf target gene, *Dusp6* (ref. 27) (Fig. 4c). Next, we assumed that the Fgf gradient modulated the BSW model by regulating the same parameters as in the mouse model—repressing *k*_4_ and boosting *k*_7_—which made the system pass through the Turing space from proximal to distal (Fig. 4c; and see Methods for the equations). When the BSW model was simulated under the influence of the Fgf gradient, a curved row of Sox9 spots was formed at a certain distance from the ectoderm (Fig. 4d). More specifically, the dynamic pattern shared two features with the observed time course: (a) it started at the anterior and posterior ends (which are also the more proximal positions) and gradually extended distally, and (b) it initially showed some connected regions of expression, which then broke up into a series of spots (compare Fig. 4d with Fig. 1b). To evaluate the role of growth in the model, we also simulated the BSW network on a static fin model, and found that without growth the *Sox9* pattern broke into spots more slowly than with growth (Supplementary Fig. 5b), suggesting that growth may contribute to reliable spot separation. In addition, both Bmp and Wnt showed strong expression in the distal region and a series of expression gaps which correspond to the spots of Sox9 (Fig. 4e), consistent with the experimental data. The relatively shallow predicted interdigital Wnt distribution was also consistent with the real expression pattern of *Wnt5b*. Therefore, the model qualitatively reproduced the expression patterns of *Sox9*, *Bmp4* and *Wnt5b* in *S. canicula* fin buds.

Our computer model reflects the normal *Sox9* patterning, but could it correctly predict the main features of experimental perturbations? A clear prediction of the model is that if Fgf signalling is reduced, the position of *Sox9* spot expressions will move closer to the distal fin edge (Fig. 4f). To test this prediction, we treated *S. canicula* embryos with the Fgf receptor inhibitor SU5402 (ref. 28), and confirmed the efficiency of inhibition by qPCR of the target gene *Dusp6*, Supplementary Fig. 5c). The resulting *Sox9* pattern showed some variability (losing its periodic form and losing expression in the anterior fin—discussed further in the legend accompanying Supplementary Fig. 5d) but it was frequently shifted distally, consistent with the prediction (Fig. 4g; Supplementary Fig. 5d for details). Thus, the row of *Sox9* expression spots appears to be positioned by Fgf signalling.

### Experimental tests for *in silico* model predictions

**Figure 5:**
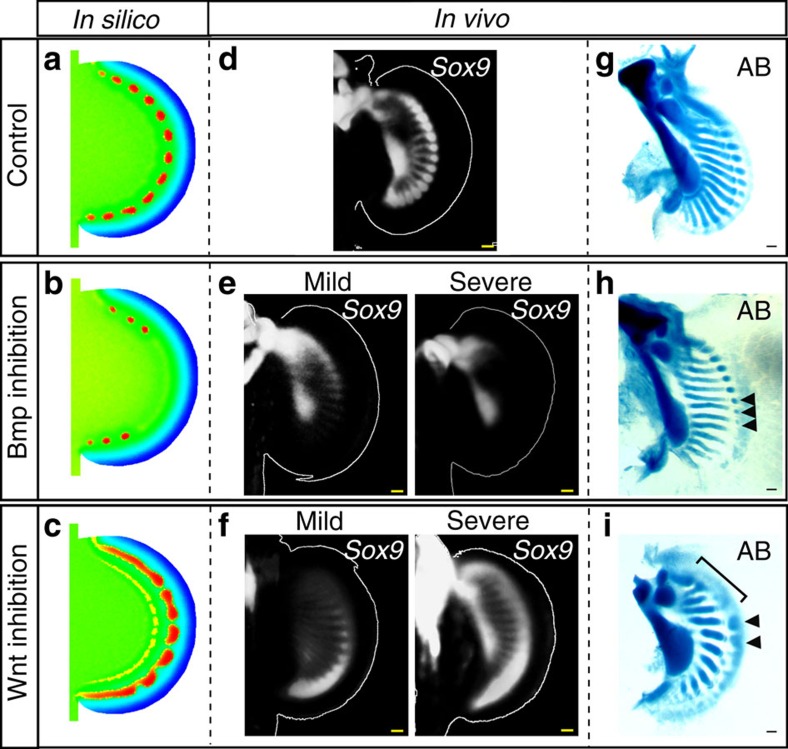
The *in silico* model can predict phenotypes of *in vivo* perturbation. (**a**) *In silico* simulation results of Sox9 (*S*) with the same parameter values as Fig. 4d. (**b**) To simulate inhibition of Bmp signalling we decreased *k*_2_ by 20% (as the LDN drug interferes with receptor binding). (**c**) To simulate inhibition of Wnt we decreased *α*_*W*_ by 50% (as the C59 drug reduces secretion of Wnt protein). (**d**–**i**) *In vivo Sox9* expression (**d**–**f**) and Alcian Blue stainings (AB; (**g**–**i**)) of pectoral fins treated with DMSO ((**d**,**g**) *n*=18/18 for *Sox9* expression, *n*=3/3 for AB), Bmp inhibitor, LDN-193189 ((**e**,**h**) *n*=6/8 for *Sox9* expression, *n*=2/2 for AB) and Wnt inhibitor, C59 ((**f**,**i**) *n*=10/10 for *Sox9* expression, *n*=3/3 for AB). Arrowheads in **h** smaller nodular elements than those in the control. Arrowheads in **i** larger nodular elements than those in the control. Bracket in **i** a continuous nodular element. Scale bars, 100 μm.

We wished to test two other molecular perturbations: inhibitions of Bmp and Wnt, to see if our model predictions would match with *in vivo* experiments. Firstly, we performed numerical simulations with decreasing values of *k*_2_, which represents inhibition of Bmp signalling. The simulation showed two features: the distal-most Sox9 spots failed to form, and those which did form were smaller (compare Fig. 5b with Fig. 5a). To carry out experimental perturbations, we treated embryos with inhibitors about 4 days before the distal *Sox9* expression appears, and checked the efficiency of inhibition by qPCR analysis and *in situ* hybridization on target genes (Supplementary Fig. 6a,b,c). Consistent with this prediction, *S. canicula* embryos treated with a Bmp inhibitor LDN-193189 (ref. 29) showed a loss of some or all of the *Sox9* spots (compare Fig. 5d and e). To assess the skeletal patterns of Bmp inhibitor-treated embryos, we carried out longer treatments on embryos and cultured them more than 1 month. Interestingly, this long-term treatment sometimes resulted in an expansion of the apical ectodermal ridge-like structure and the width of the pectoral fin buds (Supplementary Fig. 6d), suggesting that the previously reported negative effect of Bmp on the chick AER^30^ is also conserved in catshark fin buds. Cartilage staining clearly showed that the posterior nodular elements were lost and sizes of the remaining spots were smaller (black arrowheads in Fig. 5h) than in control fins (Fig. 5g), as seen in the simulation.

We next examined Wnt inhibition. In the model, this perturbation was performed by decreasing the Wnt production term. The simulated Sox9 spots became partially fused into continuous regions, and those spots which did form were larger than in the control simulation (Fig. 5c). Consistent with this *in silico* prediction, treatment with a porcupine inhibitor C59, which inhibits Wnt secretion (thus Wnt production)^31^ also resulted in a partial or complete fusion of *Sox9* into a continuous domain parallel to the distal fin edge of *S. canicula* embryos (Fig. 5f). The later cartilaginous patterns of treated fins also showed continuous or larger condensations (bracket and arrowheads in Fig. 5i)—again reflecting the simulation result. We thus found a high consistency between the model predictions and the phenotypes of *in vivo* experimental perturbations, and also a remarkable similarity in response to these inhibitors between catshark and mouse. Taken together, this suggests that the distal elements of *S. canicula* pectoral fins and mouse digits share a deeply conserved Turing system.

## Discussion

In this study, we have provided experimental and theoretical evidence that a Bmp–Sox9–Wnt Turing network represents a new example of deep homology—underlying skeletal patterning all the way from sharks to mammals. The molecular details are not identical (for example the Bmp4 ligand is the stronger candidate in the catshark, while it is BMP2 in the mouse), however the most striking feature of our results is that in both species the same basic interactions are seen between Bmp, Wnt, Sox9 and Fgf. Furthermore, the experimental perturbations of Bmp and Wnt signalling closely mirror both the model predictions and the results from mouse experiments^12^. In *S. canicula* fin buds, *Sox9* forms a spot-like pattern, which is different to the stripe-like pattern in mouse digits. However, our computer simulation reveals that the BSW network of the mouse digit patterning^12^ is also able to explain this spot pattern with just quantitative adjustments of its parameters. Therefore, relatively minor changes to the underlying deeply conserved network may be enough to trigger dramatic changes in skeletal arrangement.

Interestingly, teleosts seem not to use the BSW network (in zebrafish *sox9a* and *b* are expressed uniformly across the fin bud with no periodic pattern^32^, and *bmp2a* expression overlaps with *sox9*s (ref. 33)), and do not pattern their radials in the same manner (they produces a uniform endochondral disc, which is subsequently perforated to make the final skeletal pattern^34^). Although convergent or parallel evolution is theoretically possible—with a Turing network developing separately in cartilaginous fish and tetrapods—the most parsimonious explanation is that the BSW network was lost (or significantly altered) in the teleost lineage.

Although most components of the mouse BSW model appear to have conserved roles in the catshark (Bmp, Wnt, Sox9 and Fgf), the exception to this is the distal *Hox* genes, which play no role in our catshark model. This decoupling of the BSW network with the distal *Hox* genes is suggested by our observations and previous studies^17^,^26^ that *Hox* expression domains do not overlap with the *Sox9* expressing region significantly (Fig. 4b), and is consistent with recently published results highlighting the differences in *Hox* gene regulation between tetrapods and fish. In tetrapod limbs, *Hoxa* and *Hoxd* genes are regulated by two distinct genomic domains: a 3′ domain regulating expression in the zeugopod and a 5′ counterpart controlling autopod expression^3^,^31^. Fish fins, by contrast, do not have such strict relationship between *Hox* gene regulations and their anatomical regions. Although a bimodal regulation has been found in fish fins^3^,^6^, the expression pattern of *Hoxa13b* in zebrafish, for example, is almost uniform^35^. Similarly, misexpression of the distal *Hox* genes also causes very different results: misexpression of *Hoxd13* or *a13* in chick limb buds results in a truncation of zeugopod elements^36^,^37^, while a similar experiment in zebrafish pectoral fin buds causes an increase of cartilage condensation in the distal region^38^. Thus, much experimental data supports the idea that while the *Hox* gene regulation and anatomical modules are strictly coupled in tetrapod limb development, this modular regulation is less strict in fish fin development. Elaboration of *Hox* gene regulation, suggested by many other studies^3^,^10^,^38^,^39^, may be relevant to coupling the interaction between *Hox* genes and the BSW network in the digit patterning.

We have focused here only on the distal nodular bone formation in *S. canicula*—because it is the first periodic pattern to form in the fin bud—and thus the mechanism of the proximal stripe formation remains to be addressed. Because the distal nodular elements are each connected with a proximal stripe element in adult catshark fins, one possibility is that the proximal elements are formed just by elongation of the distal *Sox9* expression spots. Our Wnt inhibition experiments question this idea, as even when the distal *Sox9* expression becomes continuous, the stripe elements are still formed (though they are thicker and fewer in number), suggesting that formation of the stripe elements is not totally dependent on the patterning of the distal nodular elements (Fig. 5i). This semi-independent nature of the distal and proximal elements implies that additional unknown molecular controls might contribute to patterning of the proximal regions. Nevertheless, the very periodic nature of this pattern suggests that even if different molecules are involved, *Sox9* is likely patterned by a Turing-type mechanism, which may be related to the BSW model.

**Figure 6:**
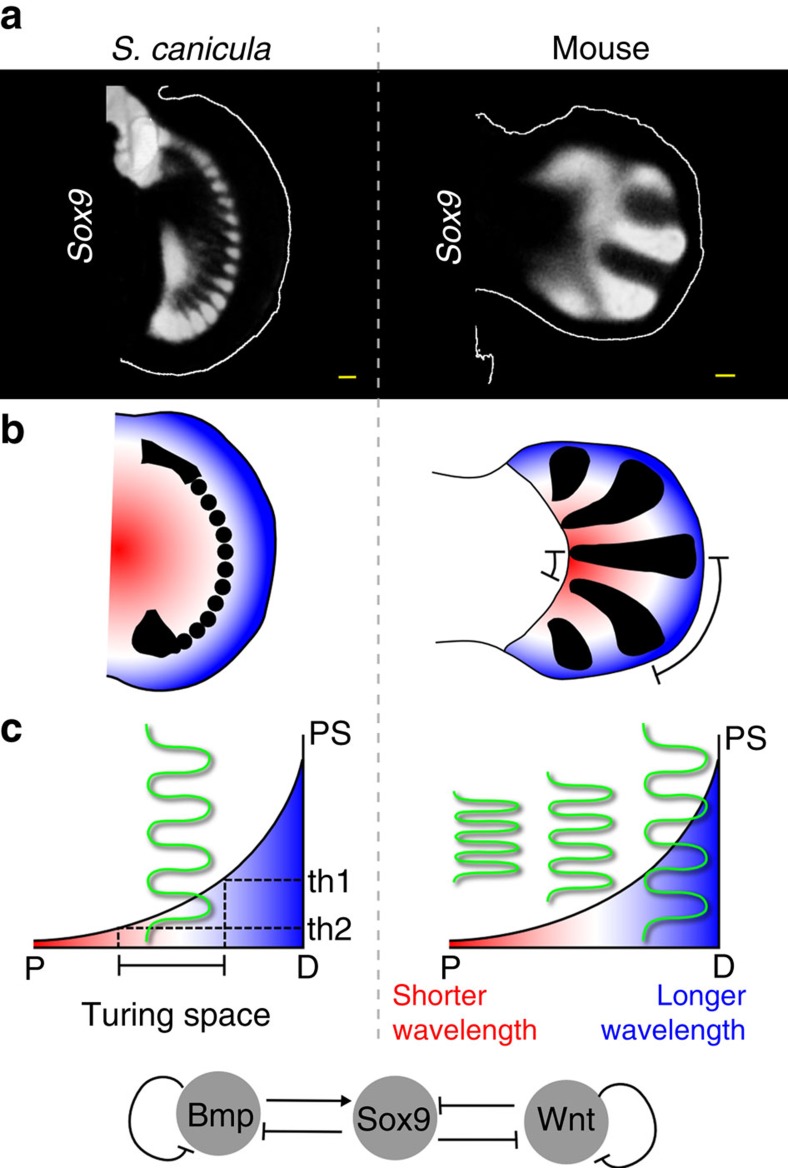
Comparison between fins and limbs. (**a**) *Sox9* expression patterns (white) in *S. canicula* pectoral fin bud at stage 30 and mouse digits at embryonic day 12. (**b**) Schematics of *Sox9* expression (black) and proximal-distal positional information represented by a smooth gradient of colours from red to blue. Bracket: the large wavelength of *Sox9* expression at the distal side of the mouse limb bud. (**c**) Two graphs illustrate the proposed difference in Fgf function between shark and mouse buds. In the case of *S. canicula* a Turing pattern (spots) can only form between the threshold values (th1 and th2). In the case of mouse a Turing pattern (stripes) forms along the whole gradient, and the Fgf signalling level instead influences the local wavelength. Space is represented on the *x* axis, and Fgf signalling (positional signal, PS) on the *y* axis. Green lines represent the Turing pattern. Bottom, the Turing network represented by this BSW model. Scale bars, 100 μm.

Finally, we propose that the changes in the fin and limb skeletal arrangement may have involved a change in the role of Fgf gradient for organizing the *Sox9* expression patterns. In *S. canicula* Fgf appears to act as a positional cue^40^—positioning the row of *Sox9* spots at a certain distance from, and therefore parallel to, the distal fin edge—while in the mouse it appears to align the digital stripes perpendicular to the distal limb edge, and to control the wavelength^41^ (Fig. 6). In the model, this dynamical difference can be explained by at least two parameters (Supplementary Fig. 7). One is the ratio between Wnt and Bmp production terms, which can change spots to stripes—also demonstrated by the Wnt inhibitor treatment in Fig. 5. The other is the inhibition of Sox9 by Wnt (*k*_3_), which affects the position of Turing space. Decreasing *k*_3_ results in a shift of Turing space to the distal edge, allowing it to form a pattern in the distal domain. In our computer model, these Wnt-related parameters are enough to change the role of Fgf gradient from positioning spots to aligning stripes. Biologically, FGF and WNT are known to have a synergetic repressive activity to *Sox9* expression^42^. Therefore, we could speculate that this synergetic activity of Wnt and Fgf might be relatively stronger in the distal mesenchyme of catshark fin bud than in the mouse digit forming region. Although the real fin-to-limb transformation must have involved more complex processes, including fin/limb shape changes, anterior–posterior patterning changes^43^, the loss of actinotrichia proteins^44^, *Hox* gene regulation^3^,^10^,^38^,^39^ and others, our simple BSW model is nevertheless able to capture some key qualitative features of this morphogenetic change.

In conclusion, our study reveals that the morphological diversity of the distal fin and limb elements can be explained as the re-organization of a Turing patterning process. It highlights how relatively small regulatory changes can lead to major re-arrangements of the skeleton, and also emphasizes the difficulty of assigning homologous relationships between the distal elements of fins and limbs.

## Methods

### Animals

Experiments were performed in accordance with guidelines for animal experiments of Tokyo Tech and CRG, and experiments involving mice were approved by animal ethics committees of CRG (JMC-07-1001P3-JS). Catshark (*Scyliorhinus canicula*) eggs were provided by A. Tweedale (Bangor university) and Station Biologique de Roscoff, France, and they were incubated at 16 °C in seawater and staged according to the standard staging system^45^. C52BL/6 (Charles River) mouse timed-pregnant females were sacrificed at different days after gestation E11.5. For *in situ* hybridization, embryos were fixed overnight in 4% paraformaldehyde in 1 M phosphate-buffered saline, dehydrated in a graded methanol series, and stored in 100% methanol at −20 °C.

### Gene isolation and *in situ* hybridization

Total RNA was extracted from stages 24 to 29 *S. canicula* embryos using an RNeasy kit (Qiagen). cDNA was synthesized by SuperScript III first strand (Invitrogen) and used as a template for PCR. To clone *S. canicula* genes, we used primers that were based on the nucleotide sequences found in the Elephant Shark Genome Project database (http://esharkgenome.imcb.a-star.edu.sg/)^46^ and SkateBase^47^,^48^ (http://skatebase.org/; Supplementary Table 1). The gene fragments were cloned into pBluescript SK—(*Bmp2*, *Bmp4* and *Dusp6*) and pCR4 (Invitrogen; *Lef1*, *Wnt5a*, *Wnt5b*, *Id3*, *Nkd1* and *Hoxa13*), respectively. The partial coding sequences for *Bmp2* (731 bp), *Bmp4* (729 bp), *Dusp6* (511 bp), *Lef1* (907 bp), *Wnt5a* (1,031 bp), *Wnt5b* (1,156 bp), *Id3* (635 bp), *Nkd1* (804 bp), *Eef1a1* (1,389 bp) and *Hoxa13* (838 bp) of *S. canicula* have been submitted to GenBank under accession numbers KT124217–KT124222, KU310672, KU310673, KU725979 respectively. Phylogenetic analysis was used to confirm the orthology of newly identified *S. canicula* genes (Supplementary Fig. 8). Amino-acid sequences were aligned using ClustalX and trimmed manually^49^. Phylogenetic trees of amino-acid sequence data sets were constructed with neighbour-joining method^50^ by MEGA5 (ref. 51). The cloned genes described above were used as templates for RNA probe synthesis. RNA probes of *Sox9* (EU241880) of *S. canicula* and mouse *Sox9* (NM_011448) respectively were synthesized as described^43^. Namely, the plasmid templates were amplified by PCR with T3/T7 or Sp6/T7 primer pairs, and transcribed by T3, T7 or Sp6 RNA polymerases (Roche). Whole-mount *in situ* hybridization was carried out with a standard protocol. Stained embryos were scanned with OPT as described^19^ and analysed with Volviewer^52^. And the colours of pictures were made grey scale with GIMP.

### Chemical treatments

*S. canicula* embryos at early stage 30 were removed from the egg shells and cultured in 6-well plate with 2∼4 ml artificial seawater containing penicillin/streptomycin (Gibco). SU5402 (Sigma), LDN-193189 (Stemgent) and C59 (Merck Millipore) were dissolved in DMSO as stock solutions. The embryos were treated with 100 μM SU5402, 50 μM LDN-193189, 20 μM C59, or indicated concentrations and 1% DMSO during 4 days and fixed for *in situ* hybridization analysis. We also analysed gene expressions 2 days after the treatments, but found no significant differences (Supplementary Fig. 6e). For qPCR analysis, the left and right pectoral fin buds of each inhibitor-treated embryos were dissected and pooled. RNA extraction was carried out with RNeasy Micro Kit (Qiagen), and cDNA synthesis was done with SuperScript III (Invitrogen). LightCycler 480 (Roche) and SYBR Green I (Roche) were used for the measurement of each gene expression amount. The qPCR primers were listed in Supplementary Table 1. Relative gene expressions were normalized by *18s rRNA* and *Eef1a1* of *S. canicula* with the following equation^53^:


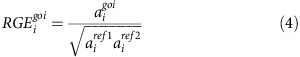


where 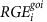 is a relative gene expression amount of the gene of interest (*goi*) in sample *i*, 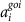 is average of gene expression amount of *goi* in sample *i*, and 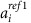 and 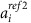 are averages of gene expression amount of *ref1* (*18S rRNA*) and ref2 (*Eef1a1*) in sample *i*, respectively. Average of gene expression amount was calculated by 
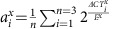
, where *x* is any gene, 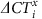 is a differential threshold cycle of gene *x* in sample *i*, and *E*^*x*^ is PCR efficiency of gene *x*. For cartilage staining, embryos were cultured with the chemicals for 20 days and additional 10–20 days with the normal artificial seawater. Before alcian blue staining, embryos were permealized by xylene and staining was carried out with a standard protocol.

### Fate map analysis

Eggs of *S. canicula* embryos around stages 26–28 were partially opened to label them with Indian ink (Pelican). To stop embryos from moving, eggs were cooled with iced seawater. Indian ink was injected into the pectoral fin buds with glass capillary, and the pictures were taken if applicable. After labelling the embryos, eggs were closed with plastic wraps and glue, and incubated in the artificial seawater containing penicillin/streptomycin (Gibco) for around 30 days. The labelled pectoral fin buds were scanned with OPT as described above. The images were manipulated with GIMP (the GNU Image Manipulation Program; http://www.gimp.org/).

### *In silico* modelling

The technique for mouse limb modelling, which was implemented with Java^22^, was applied to the 2D fin growth model. Serial pictures of *S. canicula* pectoral fin buds were taken from stage 25 to stage 32 embryos (Supplementary Fig. 3a), and their outlines were converted into spline curves. Then the shapes between each key stage were calculated by interpolation with one day temporal resolution (Supplementary Fig. 3b). Each fin shape had an independent triangle mesh implemented by gmsh^54^ (Supplementary Fig. 3c). To carry out numerical simulations in the growing fin model, each mesh transmits information of species' concentrations to the next mesh. When a mesh was deformed to match the next shape, concentration of a triangle in the deformed mesh is split into the overlapping triangles of the next mesh (as previously described^22^). This implementation also allows virtual fate map analysis (Supplementary Fig. 3d). We are happy to supply the code on request.

### Mathematical analysis and numerical simulation

Mathematical analysis of Turing space and numerical simulations were carried out in conditions previously described^12^,^55^, in particular using the linear stability analysis described in White and Gilligan^55^. We considered the following general reaction-diffusion equations for Bmp (*B*), Sox9 (*S*) and Wnt (*W*):


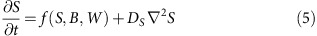



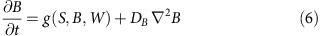



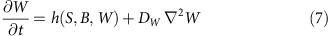


where *D*_*S*_, *D*_*B*_ and *D*_*W*_ are diffusion constants of *S*, *B* and *W* respectively. To linearize them about the steady state (S*, B*, W*), we set


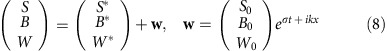


where *S*_0_, *B*_0_ and *W*_0_ are constants, *k* is the wavenumber and *σ* can either be a real number or a complex number. For |**w**| small, the equations (5)–(7), 12, 13 becomes


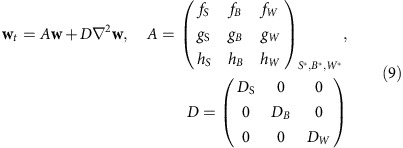


where *A* is the Jacobian matrix at the steady state, and *f*, *g* and *h* are the partial derivatives of the indicated variables. For nontrivial solutions, the *σ* is determined by the roots of the characteristic polynomial


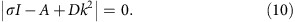


Turing instability requires


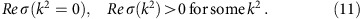


We implemented a simple linear model:


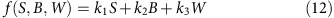



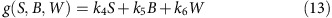



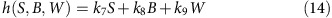


where *k*_1_ to *k*_9_ are kinetic parameters representing regulatory interactions between genes. As described previously^12^, under *D*_*S*_=0, *k*_1_=*k*_6_=*k*_8_ =0, *k*_2_>0, *k*_3_<0, *k*_5_=*k*_9_<0, we obtained the following inequality by solving (10) and (11) with Mathematica (Wolfram):


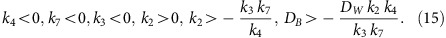


This inequality was used for determining the Turing space in Fig. 4c (parameter values were *β*=1, *k*_2_=1, *k*_3_ =1, *k*_5_ =0.1, *D*_*B*_=160, *D*_*W*_=25). For numerical simulations, partial differential equations (PDEs) were solved by PDE solver written in Java with Huen method. Time step was 0.002. The finite volume method was used to calculate the amount of diffusion between neighbouring triangles. Zero-flux boundary condition was used in all simulations. Initial conditions were set as homogeneous steady states of each species. 1% of Gaussian multiplicative noise was added at each time step. Simulations were carried out from stage 29 to stage 31. Equations (1)–(3), 2, 3 were used in Supplementary Fig. 5a. Parameter values were *β*=1, *k*_2_=1, *k*_3_=1, *k*_4_=1, *k*_7_=1, *k*_5_ =0.1, *k*_9_ =0.1, *D*_*B*_=160, *D*_*W*_=25. *α*_*B*_ and *α*_*W*_ were spatially varied from 0 to 2. The equations used in Figs 4 and 5 and Supplementary Fig. 5b are:


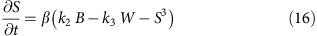



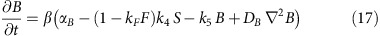



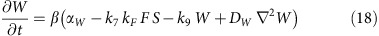


where *F* is the Fgf gradient defined below and *k*_*F*_ is a constant. Parameter values were *β*=8, *k*_2_=1, *k*_3_=3, *k*_4_=6, *k*_7_=2.4, *k*_5_=0.1, *k*_9_=0.1, *k*_*F*_=0.667, *D*_*B*_=160, *D*_*W*_=25, *α*_*B*_=0.1 and *α*_*W*_=1.2. The Fgf gradient (*F*) was created with the following equation and normalized between 0 and 1:


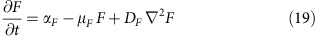


where the decay rate, *μ*_*F*_=0.1 and the diffusion constant, *D*_*F*_=600. *α*_*F*_ is a local production from the fin edge where apical ectodermal ridge is formed in *S. canicula* fin buds. For the simulations on squares in Supplementary Fig. 7, the Fgf gradient was substituted to *e*^−3*x*^ (0≤*x*≤1).

## Additional information

**Accession codes:** Sequences for newly identified genes have been deposited in the GenBank database under accession numbers KT124217 to KT124222, KU310672, KU310673, KU725979.

**How to cite this article:** Onimaru, K. *et al*. The fin-to-limb transition as the re-organization of a Turing pattern. *Nat. Commun.* 7:11582 doi: 10.1038/ncomms11582 (2016).

## Supplementary Material

Supplementary Information Supplementary Figures 1-8, Supplementary Table 1 and Supplementary References

## References

[b1] Shubin N., Tabin C. & Carroll S. Deep homology and the origins of evolutionary novelty. Nature 457, 818–823 (2009). 19212399 10.1038/nature07891

[b2] Clack J. A. The fin to limb transition: new data, interpretations, and hypotheses from paleontology and developmental biology. Annu. Rev. Earth Planet. Sci. 37, 163–179 (2009).

[b3] Woltering J. M., Noordermeer D., Leleu M. & Duboule D. Conservation and divergence of regulatory strategies at hox loci and the origin of tetrapod digits. PLoS Biol. 12, e1001773 (2014). 24465181 10.1371/journal.pbio.1001773 PMC3897358

[b4] Davis M. C., Dahn R. D. & Shubin N. H. An autopodial-like pattern of Hox expression in the fins of a basal actinopterygian fish. Nature 447, 473–476 (2007). 17522683 10.1038/nature05838

[b5] Johanson Z. . Fish fingers: digit homologues in Sarcopterygian fish fins. J. Exp. Zool. Part B Mol. Dev. Evol. 308, 757–768 (2007). 10.1002/jez.b.21197 17849442

[b6] Gehrke A. R. . Deep conservation of wrist and digit enhancers in fish. Proc. Natl Acad. Sci. USA 112, 803–808 (2015). 25535365 10.1073/pnas.1420208112 PMC4311833

[b7] Boisvert C. A., Mark-Kurik E. & Ahlberg P. E. The pectoral fin of Panderichthys and the origin of digits. Nature 456, 636–638 (2008). 18806778 10.1038/nature07339

[b8] Coates M. The evolution of paired fins. Theor. Biosci. 122, 266–287 (2003).

[b9] Davis M. C., Shubin N. H. & Daeschler E. B. A new specimen of *Sauripterus taylori* (Sarcopterygii, Osteichthyes) from the Famennian Catskill Formation of North America. J. Vert. Paleontol. 24, 26–40 (2004).

[b10] Schneider I. . Appendage expression driven by the Hoxd Global Control Region is an ancient gnathostome feature. Proc. Natl Acad. Sci. USA 108, 12782–12786 (2011). 21765002 10.1073/pnas.1109993108 PMC3150877

[b11] Sheth R. . Hox genes regulate digit patterning by controlling the wavelength of a Turing-type mechanism. Science 338, 1476–1480 (2012). 23239739 10.1126/science.1226804 PMC4486416

[b12] Raspopovic J., Marcon L., Russo L. & Sharpe J. Digit patterning is controlled by a Bmp-Sox9-Wnt Turing network modulated by morphogen gradients. Science 345, 566–570 (2014). 25082703 10.1126/science.1252960

[b13] Newman S. A. & Frisch H. L. Dynamics of skeletal pattern formation in developing chick limb. Science 205, 662–668 (1979). 462174 10.1126/science.462174

[b14] Miura T., Shiota K., Morriss-Kay G. & Maini P. K. Mixed-mode pattern in Doublefoot mutant mouse limb-Turing reaction-diffusion model on a growing domain during limb development. J. Theor. Biol. 240, 562–573 (2006). 16364368 10.1016/j.jtbi.2005.10.016

[b15] Zhu J., Zhang Y. T., Alber M. S. & Newman S. A. Bare bones pattern formation: A core regulatory network in varying geometries reproduces major features of vertebrate limb development and evolution. PLoS ONE 5, (2010). 10.1371/journal.pone.0010892 PMC2878345 20531940

[b16] Turing A. M., Transactions P., Society R., Sciences B. & Turing B. Y. A. M. The chemical basis of morphogenesis. Philos. Trans. R. Soc. B 237, 37–72 (1952).

[b17] Freitas R., Zhang G. & Cohn M. J. Biphasic Hoxd gene expression in shark paired fins reveals an ancient origin of the distal limb domain. PLoS ONE 2, e754 (2007). 17710153 10.1371/journal.pone.0000754 PMC1937022

[b18] Renz A. J., Meyer A. & Kuraku S. Revealing less derived nature of cartilaginous fish genomes with their evolutionary time scale inferred with nuclear genes. PLoS ONE 8, e66400 (2013). 23825540 10.1371/journal.pone.0066400 PMC3692497

[b19] Sharpe J. . Optical projection tomography as a tool for 3D microscopy and gene expression studies. Science 296, 541–545 (2002). 11964482 10.1126/science.1068206

[b20] Benazet J.-D. . Smad4 is required to induce digit ray primordia and to initiate the aggregation and differentiation of chondrogenic progenitors in mouse limb buds. Development 139, 4250–4260 (2012). 23034633 10.1242/dev.084822

[b21] Atcha F. A., Munguia J. E., Li T. W. H., Hovanes K. & Waterman M. L. A new beta-catenin-dependent activation domain in T cell factor. J. Biol. Chem. 278, 16169–16175 (2003). 12582159 10.1074/jbc.M213218200

[b22] Marcon L., Arqués C. G., Torres M. S. & Sharpe J. A computational clonal analysis of the developing mouse limb bud. PLoS Comp. Biol. 7, e1001071 (2011). 10.1371/journal.pcbi.1001071 PMC3037386 21347315

[b23] Muneoka K., Wanek N. & Bryant S. V. Mammalian limb bud development: *in situ* fate maps of early hindlimb buds. J. Exp. Zool. 249, 50–54 (1989). 2926361 10.1002/jez.1402490110

[b24] Nomura N., Yokoyama H. & Tamura K. Altered developmental events in the anterior region of the chick forelimb give rise to avian-specific digit loss. Dev. Dyn. 243, 741–752 (2014). 24616028 10.1002/dvdy.24117

[b25] Tanaka M. . Fin development in a cartilaginous fish and the origin of vertebrate limbs. Nature 416, 527–531 (2002). 11932743 10.1038/416527a

[b26] Sakamoto K. . Heterochronic shift in Hox-mediated activation of Sonic hedgehog leads to morphological changes during fin development. PLoS ONE 4, e5121 (2009). 19365553 10.1371/journal.pone.0005121 PMC2664896

[b27] Eblaghie M. C. . Negative feedback regulation of FGF signaling levels by Pyst1/MKP3 in chick embryos. Curr. Biol. 13, 1009–1018 (2003). 12814546 10.1016/s0960-9822(03)00381-6

[b28] Mohammadi M. . Structures of the tyrosine kinase domain of fibroblast growth factor receptor in complex with inhibitors. Science 276, 955–960 (1997). 9139660 10.1126/science.276.5314.955

[b29] Yu P. B. . BMP type I receptor inhibition reduces heterotopic ossification. Nat. Med. 14, 1363–1369 (2008). 19029982 10.1038/nm.1888 PMC2846458

[b30] Pizette S. & Niswander L. BMPs negatively regulate structure and function of the limb apical ectodermal ridge. Development 126, 883–894 (1999). 9927590 10.1242/dev.126.5.883

[b31] Proffitt K. D. . Pharmacological inhibition of the Wnt acyltransferase PORCN prevents growth of WNT-driven mammary cancer. Cancer Res. 73, 502–507 (2013). 23188502 10.1158/0008-5472.CAN-12-2258

[b32] Yan Y.-L. . A pair of Sox: distinct and overlapping functions of zebrafish sox9 co-orthologs in craniofacial and pectoral fin development. Development 132, 1069–1083 (2005). 15689370 10.1242/dev.01674

[b33] Thisse B. & Thisse C. High throughput expression analysis of ZF-models consortium clones. ZFIN Direct Data Submission (2005). Available at http://zfin.org/cgi-bin/webdriver?MIval=aa-pubview2.apg&OID=ZDB-PUB-051025-1.

[b34] Grandel H. & Schulte-Merker S. The development of the paired fins in the Zebrafish (Danio rerio). Mech. Dev. 79, 99–120 (1998). 10349624 10.1016/s0925-4773(98)00176-2

[b35] Ahn D. & Ho R. K. Tri-phasic expression of posterior Hox genes during development of pectoral fins in zebrafish: implications for the evolution of vertebrate paired appendages. Dev. Biol. 322, 220–233 (2008). 18638469 10.1016/j.ydbio.2008.06.032

[b36] Goff D. J. & Tabin C. J. Analysis of Hoxd-13 and Hoxd-11 misexpression in chick limb buds reveals that Hox genes affect both bone condensation and growth. Development 124, 627–636 (1997). 9043077 10.1242/dev.124.3.627

[b37] Yokouchi Y. . Misexpression of HoxA-13 induces cartilage homeotic transformation and changes cell adhesiveness in chick limb buds. Genes Dev. 9, 2509–2522 (1995). 7590231 10.1101/gad.9.20.2509

[b38] Freitas R., Gómez-Marín C., Wilson J. M., Casares F. & Gómez-Skarmeta J. L. Hoxd13 contribution to the evolution of vertebrate appendages. Dev. Cell 23, 1219–1229 (2012). 23237954 10.1016/j.devcel.2012.10.015

[b39] Amemiya C. T. . The African coelacanth genome provides insights into tetrapod evolution. Nature 496, 311–316 (2013). 23598338 10.1038/nature12027 PMC3633110

[b40] Wolpert L. Positional information and the spatial pattern of cellular differentiation. J. Theor. Biol. 25, 1–47 (1969). 4390734 10.1016/s0022-5193(69)80016-0

[b41] Green J. B. A. & Sharpe J. Positional information and reaction-diffusion: two big ideas in developmental biology combine. Development 142, 1203–1211 (2015). 25804733 10.1242/dev.114991

[b42] ten Berge D., Brugmann S. A., Helms J. A. & Nusse R. Wnt and FGF signals interact to coordinate growth with cell fate specification during limb development. Development 135, 3247–3257 (2008). 18776145 10.1242/dev.023176 PMC2756806

[b43] Onimaru K. . A shift in anterior–posterior positional information underlies the fin-to-limb evolution. eLIFE 4, e07048 (2015). 10.7554/eLife.07048 PMC4538735 26283004

[b44] Zhang J. . Loss of fish actinotrichia proteins and the fin-to-limb transition. Nature 466, 234–237 (2010). 20574421 10.1038/nature09137

[b45] Ballard W. W., Mellinger J. & Lechenault H. A series of normal stages for development of Scyliorhinus canicula, the lesser spotted dogfish (Chondrichthyes: Scyliorhinidae). J. Exp. Zool. 267, 318–336 (1993).

[b46] Venkatesh B. . Elephant shark genome provides unique insights into gnathostome evolution. Nature 505, 174–179 (2014). 24402279 10.1038/nature12826 PMC3964593

[b47] Wyffels J. . SkateBase, an elasmobranch genome project and collection of molecular resources for chondrichthyan fishes. F1000Res. 3, 191 (2014). 25309735 10.12688/f1000research.4996.1 PMC4184313

[b48] Wang Q. . Community annotation and bioinformatics workforce development in concert--Little Skate Genome Annotation Workshops and Jamborees. Database 2012, bar064 (2012). 22434832 10.1093/database/bar064 PMC3308154

[b49] Thompson J. D., Gibson T. J., Plewniak F., Jeanmougin F. & Higgins D. G. The CLUSTAL_X windows interface: flexible strategies for multiple sequence alignment aided by quality analysis tools. Nucleic Acids Res. 25, 4876–4882 (1997). 9396791 10.1093/nar/25.24.4876 PMC147148

[b50] Saitou N. & Nei M. The neighbor-joining method: a new method for reconstructing phylogenetic trees. Mol. Biol. Evol. 4, 406–425 (1987). 3447015 10.1093/oxfordjournals.molbev.a040454

[b51] Tamura K. . MEGA5: molecular evolutionary genetics analysis using maximum likelihood, evolutionary distance, and maximum parsimony methods. Mol. Biol. 28, 1530–1534 (2011). 10.1093/molbev/msr121 PMC3203626 21546353

[b52] Lee K. . Visualizing plant development and gene expression in three dimensions using optical projection tomography. Plant Cell 18, 2145–2156 (2006). 16905654 10.1105/tpc.106.043042 PMC1560903

[b53] Hellemans J., Mortier G., De P. A., Speleman F. & Vandesompele J. qBase relative quantification framework and software for management and automated analysis of real-time quantitative PCR data. Genome Biol. 8, R19 (2007). 17291332 10.1186/gb-2007-8-2-r19 PMC1852402

[b54] Geuzaine C. & Remacle J.-F. Gmsh: a three-dimensional finite element mesh generator with built-in pre- and post-processing facilities. Int. J. Numer. Methods Eng 79, 1309–1331 (2009).

[b55] White K. A. J. & Gilligan C. A. Spatial heterogeneity in three-species, plant-parasite-hyperparasite, systems. Phil.Trans. R. Soc. Lond. B 353, 543–557 (1998).

