# Supplementary material for: The fin-to-limb transition as the re-organization of a Turing pattern
Source: Nat Commun. 2016 May 23;7:11582. doi: 10.1038/ncomms11582 (PMC4879262; doi:10.1038/ncomms11582)
Supplement: Supplementary Information — Supplementary Figures 1-8, Supplementary Table 1 and Supplementary References [file ncomms11582-s1.pdf]

Supplementary Figures

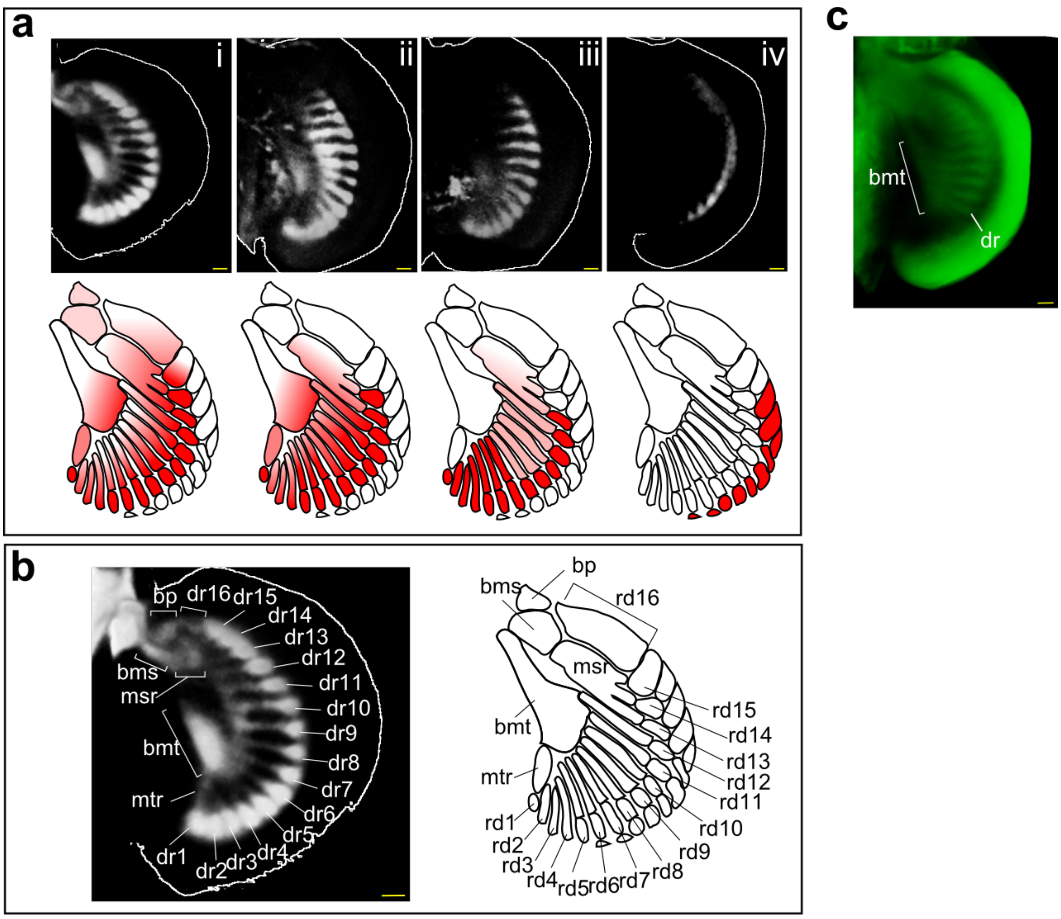

Supplementary Figure 1 | Detailed description of *Sox9* expression pattern. a, OPT scans of *Sox9* expression in *S. canicula* pectoral fin buds at later stages (stage 30~31). Each of the spots starts to extend its expression towards the proximal part of the fin, forming proximal stripy radials (a-i-iii). Spot pattern of *Sox9* expression corresponding to the most distal nodular elements appears stage 31 (a-iv). At this stage, cellular condensation seems to have already started (see **c**). **b**, the detailed annotation of *Sox9* expression of **a**-i and an adult skeletal pattern. bp, basal propterygium; bms, basal

mesopterygium; bmt, basal metapterygium; msr, mesopterygial radial; mtr, metapterygial radial; dr1-16, distal radials. **c**, nuclear staining by propidium iodide of **a**-iv. Scale bars: 100 µm.

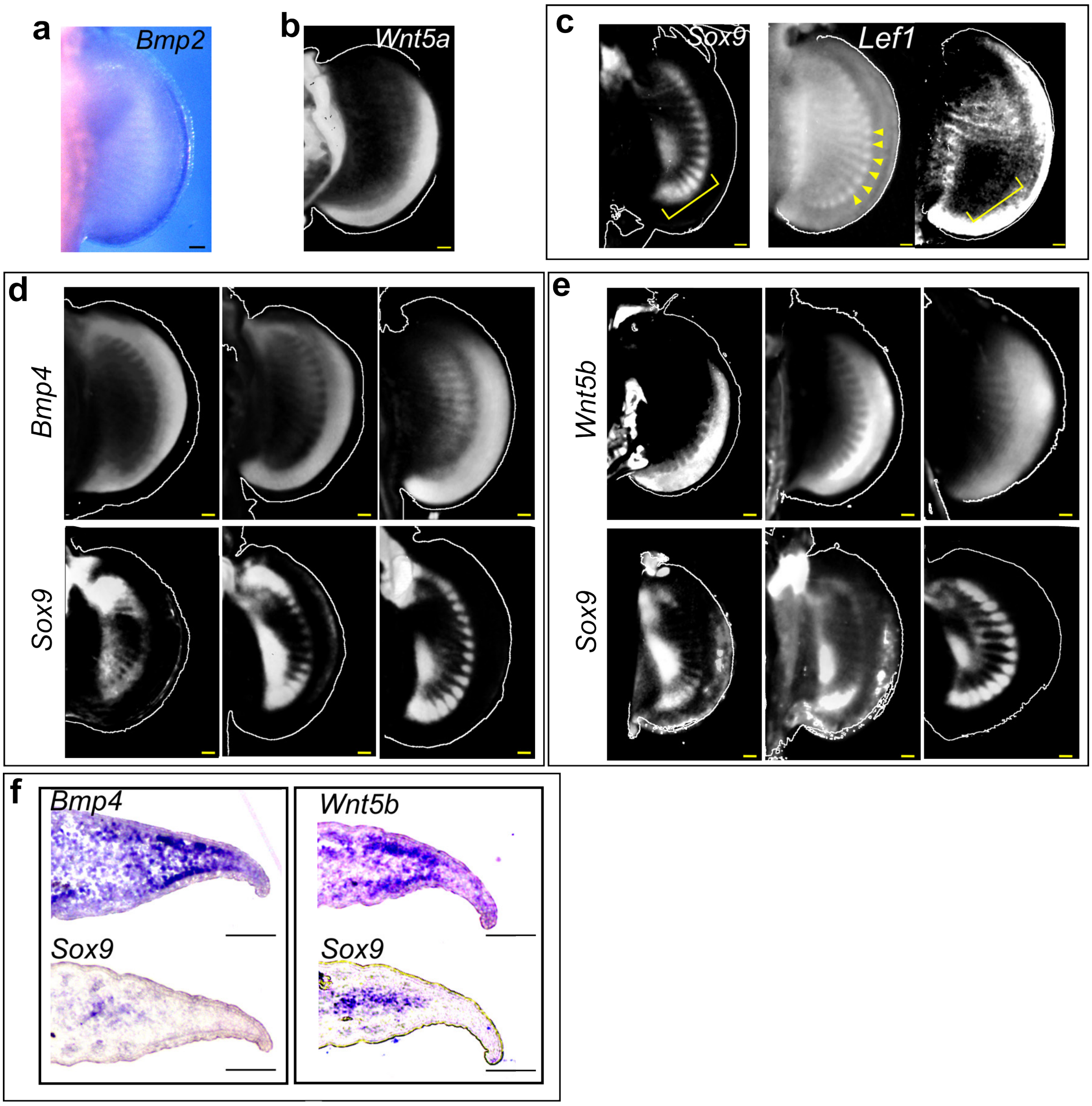

Supplementary Figure 2 | Expressions of Bmp and Wnt related genes in *S. canicula* pectoral fin buds. a, Bmp2 expression. **b,** OPT scan of *Wnt5a* expression, which only showed a smooth gradient from distal to proximal, contrary to *Wnt5b*. **c**, OPT scans of left (*Sox9*) and right (*Lef1*) pectoral fin bud from one *S. canicula* embryo. Because *Lef1* expression was also present in muscle progenitors (arrowheads), this part of the 3D dataset was digitally dissected away in the right panel. *Lef1* had a shallow complementary

expression with *Sox9* especially in the posterior part of pectoral fin buds (compare brackets). **d, e,** Sequential expressions of *Bmp4* and *Wnt5b* in right pectoral fins. Bottom panels are *Sox9* expressions of the left pectoral fins from the same embryos of panels above. **f,** *Bmp4*, *Wnt5b* and *Sox9* expressions in serial transverse sections of anterior (left panel) or posterior (right panel) part of *S. canicula* pectoral fin buds**.** Top and bottom panels are neighbouring sections from one fin bud. Scale bars: 100 µm.

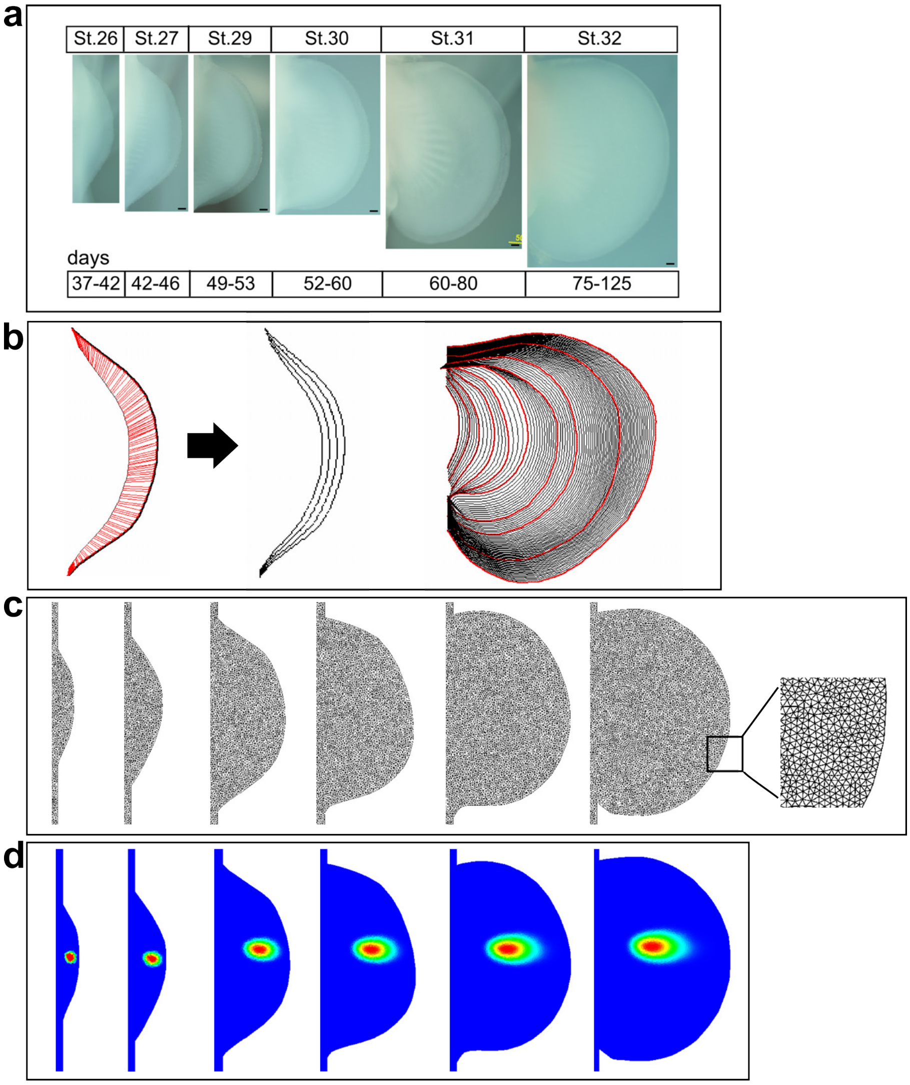

Supplementary Figure 3 | Creating the fin growth model. **a**, Dorsal views of *S. canicula* pectoral fin buds from stage 26 to 32. Anterior is to the top. Outlines of these fin buds were used for the fin growth model. **b**, Interpolation of shape gaps between the fin outlines. Left, an example of interpolation. Outlines of two neighboring stages were aligned manually, and corresponding points between the two outlines were inferred (red lines in the most left figure in **b**). Middle, interpolated shapes between the two outlines of the fin shapes in the left figure. Right, the full set of interpolated shapes. **c**, The fin

growth model from stage 26 to stage 31. Each shape has a fine triangular mesh. See magnified view in the most right fin. **d**, An example of virtual fate map analysis. A few triangular elements are labeled with a red dye (probability equal to one) at early stage (the most left fin) and its fate of the dye is simulated using the sequence of deformations and interpolations as seen in red to blue colors (high to low probability of dye concentration). Scale bars: 100 µm.

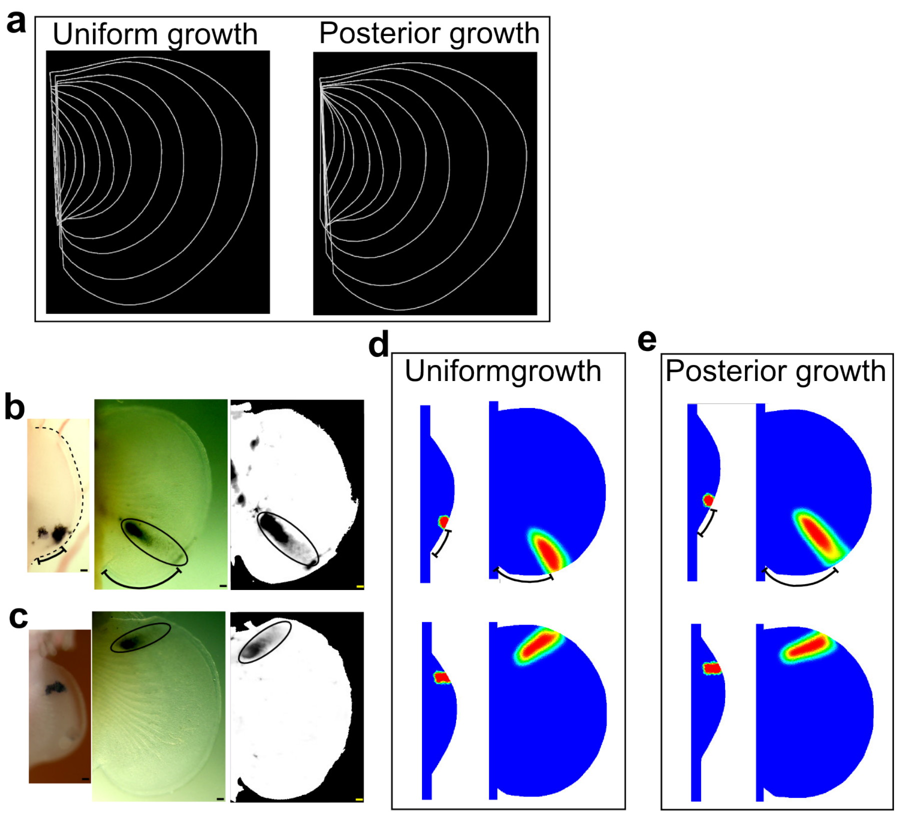

Supplementary Figure 4 | Fate map analysis. a, Examples of fin shape alignments. The fin shapes were aligned at the center (uniform growth) or at the anterior (posterior growth). **b**, **c**, Fate map analyses of *S. canicula* pectoral fin buds with Indian ink. Left panels, fin buds at stage 26~7 immediately after the labeling. Middle panels, the result fin buds after 30 (**b**) and 28 (**c**) days (stage 31), respectively. Right panels, OPT scans of the result fin buds. Circles indicate the distribution of Indian ink. Brackets indicate the distance between the labeled cells and the posterior (**b**) or anterior (**c**) margins of the fin buds. Note that the distance from the posterior margin almost triples in the resulting fin buds in **b**. **d**, **e**, Virtual fate map analyses produces slightly different predicted growth patterns, depending on the fin growth models aligned at the central (uniform growth) and the

anterior (posterior growth). As indicated by brackets, the posterior growth model can reproduce the tripling of the distance from the posterior margin of fin bud as seen in the fate map analyses, but the uniform growth model cannot. Scale bars: 100 µm.

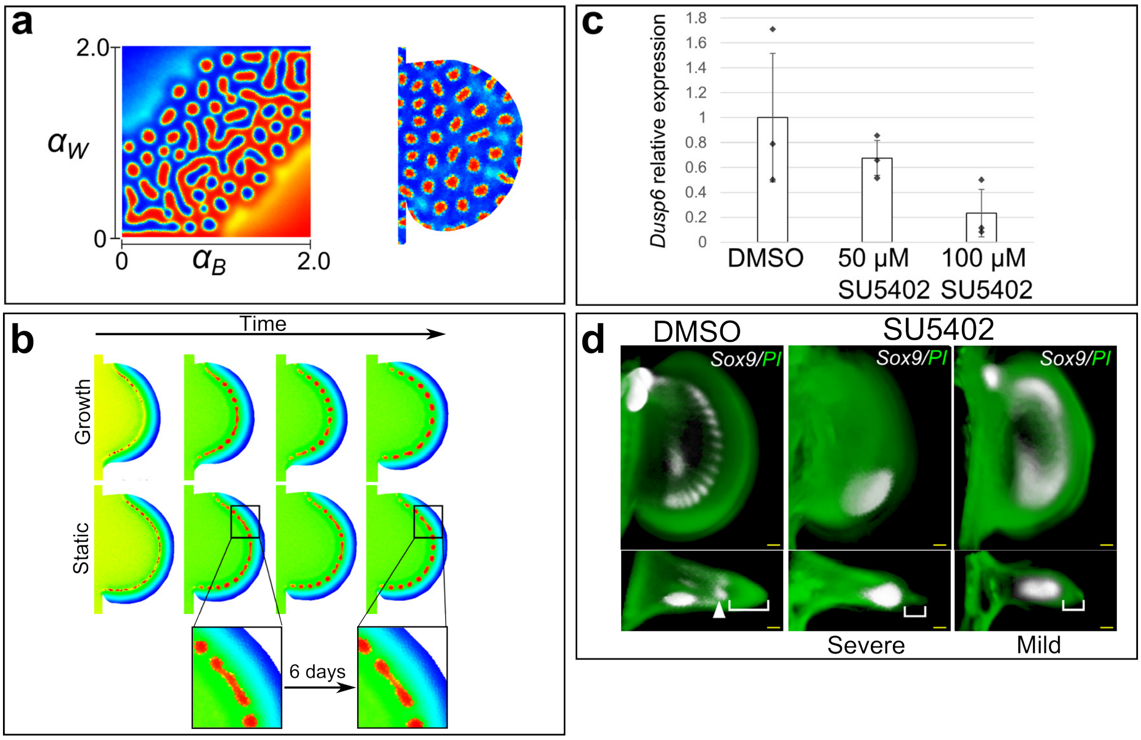

Supplementary Figure 5 | Model simulations and FGF inhibition analyses in *S. canicula* pectoral fin buds. **a,** Left panel, a numerical simulation of Sox9 expression in a square domain, with spatially-varying production terms of Bmp (α_B) and Wnt (α_W). Right figure, a simulation in the fin growth model with α_B=1.2 and α_W=0.1, with no other spatial constraints on the Turing pattern, resulting in a uniform spot pattern (see Methods for other parameters). **b,** Top: the simulation of the BSW model in the growing fin-bud domain. Bottom: the same model but simulated in a static, non-growing fin bud domain. **c**, qPCR analysis of *Dusp6* expression in DMSO and SU5402 treated fin buds. Each dot represents a gene expression measure on the pectoral fin buds of an individual embryo (n=3 for each treatment). Efficient downregulation of the *Dusp6* gene is observed with

100 µM SU5402 but not 50 µM. Error bars are s. d. **d**, OPT scans of *Sox9* expression (white) stained with propidium iodide (green) in DMSO or Fgf inhibitor, SU5402 treated pectoral fin buds (left, severe phenotype; *n*=4/12; right, mild phenotype; *n*=3/12). Dorsal views; anterior is to the top. Lower panels: virtual transverse sections (dorsal is to the top; distal is to the right). Arrowhead: spot expression of *Sox9.* Bracket: distance from the distal margin of the fin buds. The overall pattern of *Sox9* was quite variable – presumably because Fgf has multiple roles, such as a positive feed back loop with Shh [1,2] and also Wnt[3]. However, the distal shift was consistent across all samples. The feedback loop with Shh probably explains why the posterior part of the *Sox9* expression was more robust against the perturbation than the anterior region. The loss of periodicity of the *Sox9* pattern (it routinely became continuous) may be due to a downregulation of Wnt (similar to the Wnt inhibition experiments shown in Fig. 3) consistent to the known synergistic effect between Fgf and Wnt[4]. Scale bars: 100 µm.

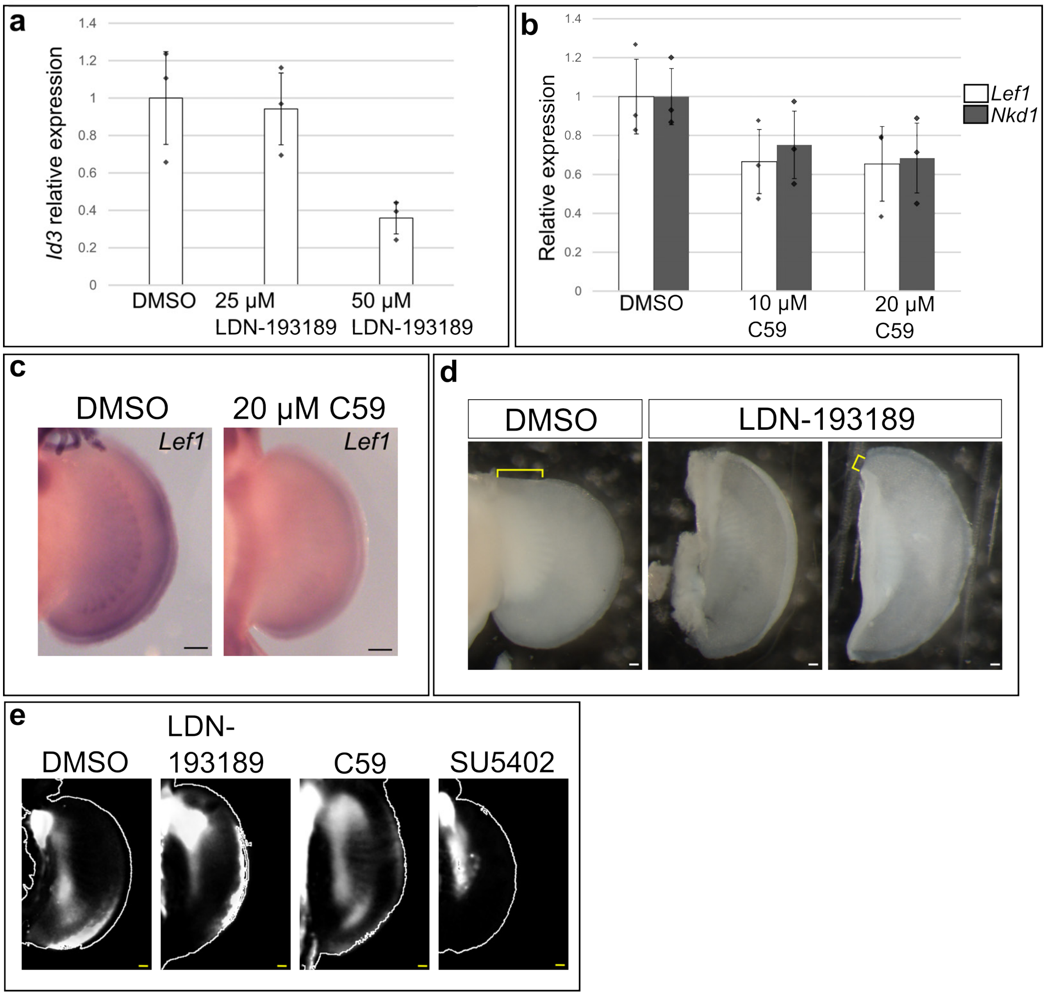

Supplementary Figure 6 | Bmp and Wnt inhibition analysis. a, b, qPCR analysis of *Id3* (**a**), *Lef1* and *Nkd1* (**b**) expression in Bmp (**a**) and Wnt (**b**) inhibitor treated pectoral fin buds, respectively. Each dot represents a gene expression measure on the pectoral fin buds of an individual *S. canicula* embryo (*n*=3 for each treatment). Error bars are s. d. Note that a Bmp target gene, *Id3*[5] expression is efficiently downregulated in 50 μM LDN-193189 treatment but not in 25 μM (**a**). The expression of Wnt target genes, *Lef1* and *Nkd1*[6] is moderately downregulated in both concentrations (**b**). **c,** Whole-mount *in situ* hybridisation of *Lef1* on DMSO (*n*=2/2) and 20 μM C59 (*n*=3/4) treated pectoral fin buds

to verify the qPCR analysis. Note that clear downregulation of *Lef1* is observed in the C59 treated pectoral fin bud. **d,** *S. canicula* pectoral fin buds around 20 days after DMSO (*n*=1) and Bmp inhibitor treatments (*n*=2/2). Brackets indicate anterior fin edges where the AER-like structure does not form. In the Bmp inhibitor treated fin buds, such AER-free anterior fin edges shrunk or did not form, and the fin width along the anterior-posterior axis was broader than the control. **e,** OPT scans of *Sox9* expressions in *S. canicula* pectoral fin buds 2 days after treatments of each inhibitors. Scale bars: 100 µm.

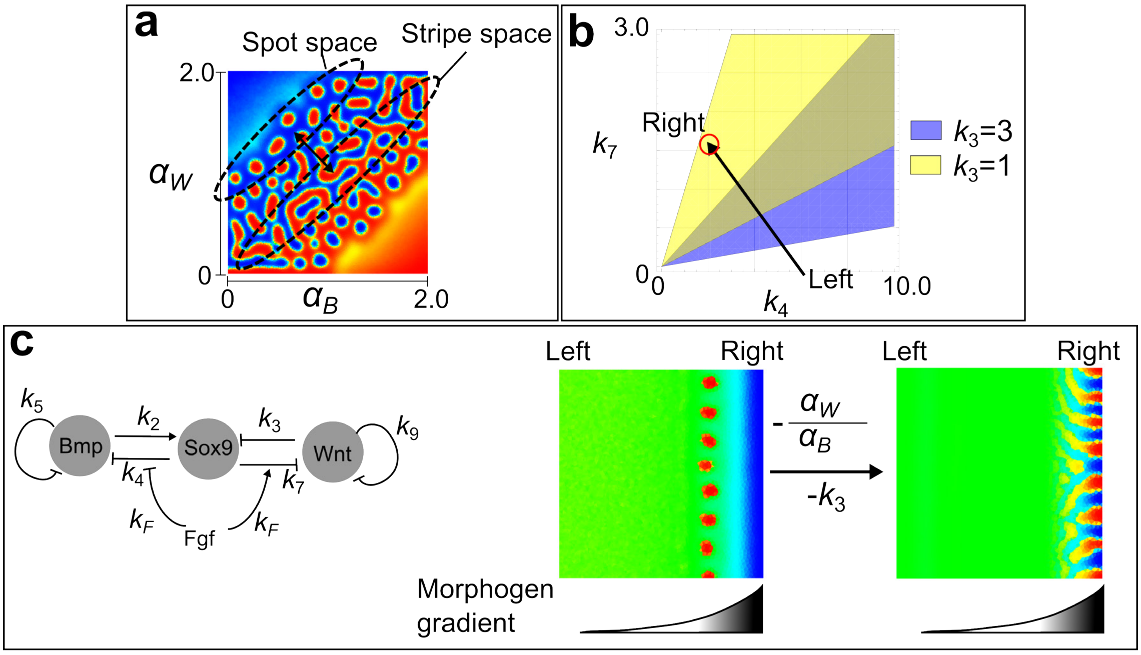

Supplementary Figure 7 | Illustration of how the same circuit can explain both the mouse and the catshark skeletal pattern. a, The same simulation as in Supplementary Fig. 5a, illustrating how different parameter values can produce either spots or stripes. The spot pattern becomes a stripe pattern when the ratio of Wnt production to Bmp production decreases. **b,** A 2D parameter space for k_4 and k_7. Yellow and blue domains are Turing spaces with k_3=1 and k_3=3, respectively. The arrow indicates the effect of the Fgf morphogen to the BSW network in **c**. The red circle indicates the point of the arrow with highest Fgf levels (i.e. the right edge of the squares in **c**). Note that while the tip of the arrow is out of Turing space (blue) when k_3=3, it is in the Turing space (yellow) when k_3=1, meaning that Turing space is shifted to the right edge of the square in **c**. **c,** parameters (left panel) and simulations (right panels) of the BSW network. The

morphogen (Fgf) diffuses from the right edge of the squares. A row of spots parallel to the right edge (left) represents the *S. canicula* case. Stripes perpendicular to the right edge (right panel) represents the mouse digit state. Decreasing the ratio of α_W to α_B and k_3 (or the ratio of k_3 to k_2) from the *S. canicula* case results in a shift of Turing space to the right edge, and also a change from spots to stripes, representing the mouse case. Scale bars: 100 µm.

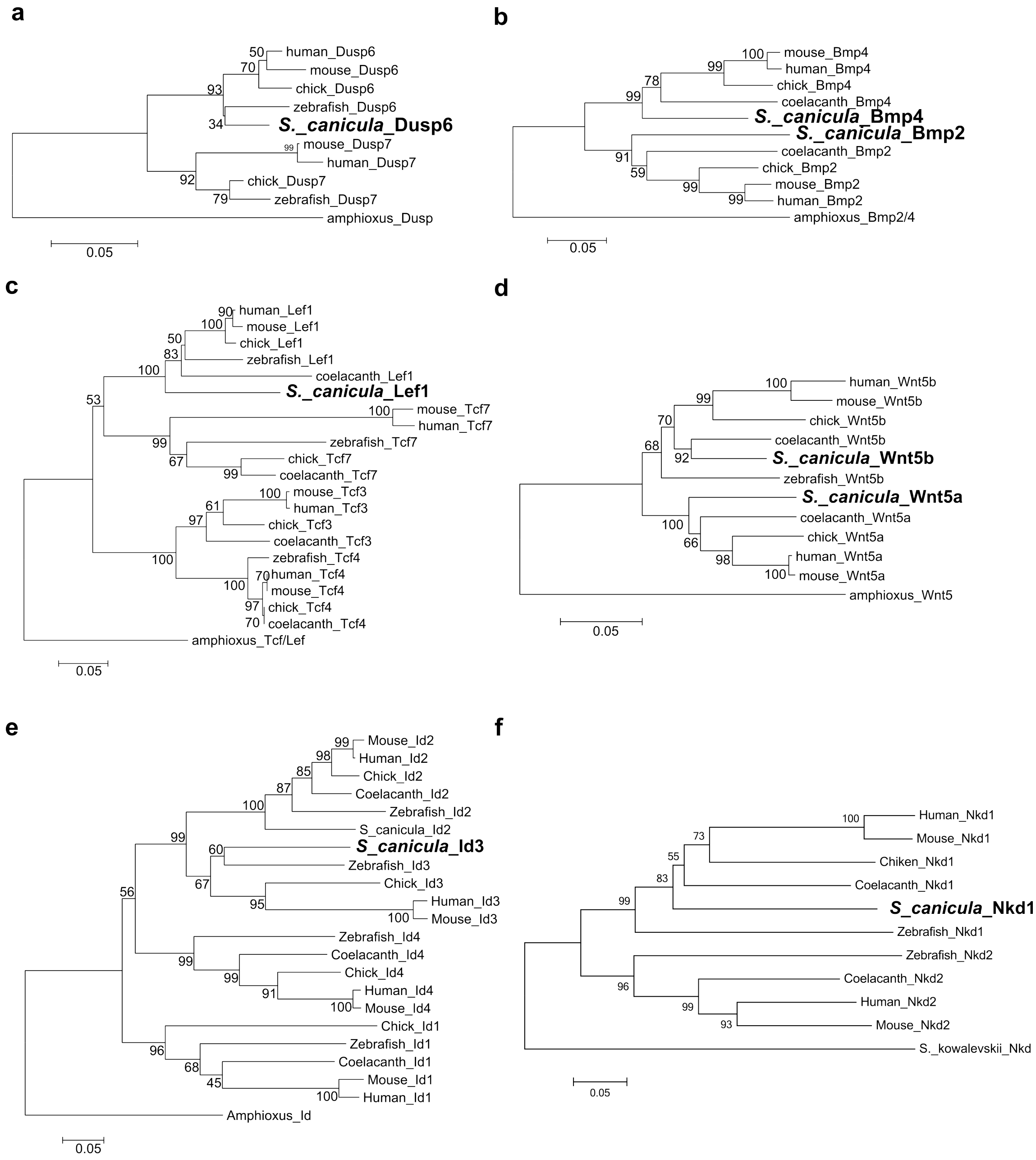

Supplementary Figure 8 | Molecular phylogenetic trees of relevant *S. canicula* genes.

a-f, Phylogenetic trees for Dusp6 (**a**), Bmp (**b**), Lef1 (**c**), Wnt5a/b (**d**), Id3 (**e**) and Nkd1 (**f**) were generated from the amino acid sequences of C-terminal domain (**a, b**), Ctnnb-1 binding domain and HMG-box (**c**) and whole sequence (**d-f**). The neighbour-joining

method was used for constructing the trees. Pairwise deletion was used for Id genes. The numbers at nodes indicate bootstrap probabilities with 1000 replicates.

Supplementary Table

Supplementary Table 1 | Primer list

Gene	Forward primer	Reverse primer	Experiment	Sequences	Product length (bp)
Bmp2	5′- GGCCGAGCTGAGGATCTTCCG -3′	5′- TCCACGACCATATCTTGGTAG -3′	Cloning	AAVX01033842.1	731
Bmp4	5′- TGATTCCTGGTAACCGAATGC -3′	5′- TCCACATAGAGCGAGTGCCTC -3′	Cloning	AAVX01001906.1	729
Dusp6	5′- CTATCTGATGCAGAAGCTGAACC -3′	5′- AGAGTGTGAACACTTCACGTGAC -3′	Cloning	SSC-transcript-ctg15246	511
Hoxa13	5′- AACATGGATGGATTTTCAGGAGGGAAC -3′	5′- AAGTCCGCACGTTTCTCGGATTGTCGC -3′	Cloning	SSC-transcript-ctg30266	838
Id3	5′- CGACATGAACGACTGCTACTGTAAG -3′	5′- TAACACTCTGATCCGAGTGTCCTGG -3′	Cloning	SSC-transcript-ctg70205	635
Lef1	5′- ACGACGAGATGATCTCGTTCAAGG -3′	5′- TACTTTGCTTGCTCTTCACGAGAC -3′	Cloning	SSC-transcript-ctg21954	907
Wnt5a	5′- CTGCAAACAGGTCCTAGTGGAAG -3′	5′- TGCAAACGAATTGATCAACCACC -3′	Cloning	SSC-transcript-ctg15761	1031
Wnt5b	5′- ATGCAGAGGACGGTGGCAGGAC -3′	5′- GTTATTTGCAAACAAACTGATC -3′	Cloning	SSC-transcript-ctg94055	1156
18S rRNA	5′- GCATTCGTATTGTGCCGCTAGAGGTG -3′	5′- AAGTTTCAGCTTTGCAACCATACTCC -3′	qPCR	GQ395808.1	259
Dusp6	5′- CACCAACCTGGACGTGCTGGAGGAG -3′	5′- TTCTTCATCTTCACAATGTCGTAGG -3′	qPCR	SSC-transcript-ctg15246	315
Eef1a1	5′- TGGACTCTGGCAAATCCACTACCACTG -3′	5′- CCAGAGTGTAGGCTAGCAAGGCATG -3′	qPCR	SSC-transcript-ctg24381	384
Id3	5′- CGACATGAACGACTGCTACTGTAAG -3′	5′- AACTATCTCCTTCACTGGACAGTTC -3′	qPCR	SSC-transcript-ctg70205	221
Lef1	5′- ATCCTATGGTTCCTGGTAACCATC -3′	5′- TACTTTGCTTGCTCTTCACGAGAC -3′	qPCR	SSC-transcript-ctg21954	304

Nkd1	5′- AACTGCCATCGTCACTGTGTGGATG -3′	5′- GGCTGTGTCCTGGATCCATAGTTTG -3′	qPCR	SSC-transcript-ctg18820	228

Supplementary references

1. Laufer, E., Nelson, C. E., Johnson, R. L., Morgan, B. A. & Tabin, C. Sonic hedgehog and Fgf-4 act through a signaling cascade and feedback loop to integrate growth and patterning of the developing limb bud. *Cell* **79,** 993–1003 (1994).

2. Niswander, L., Jeffrey, S., Martin, G. R. & Tickle, C. A positive feedback loop coordinates growth and patterning in the vertebrate limb. *Nature* **371,** 609–612 (1994).

3. Kawakami, Y. *et al.* WNT signals control FGF-dependent limb initiation and AER induction in the chick embryo. *Cell* **104,** 891–900 (2001).

4. ten Berge, D., Brugmann, S. A., Helms, J. A. & Nusse, R. Wnt and FGF signals interact to coordinate growth with cell fate specification during limb development. *Development* **135,** 3247–3257 (2008).

5. Hollnagel, A., Oehlmann, V., Heymer, J., Rüther, U. & Nordheim, A. Id genes are direct targets of bone morphogenetic protein induction in embryonic stem cells. *J. Biol. Chem.* **274,** 19838–19845 (1999).

6. Ishikawa, A. *et al.* Mouse Nkd1, a Wnt antagonist, exhibits oscillatory gene

expression in the PSM under the control of Notch signaling. *Mech. Dev.* **121,** 1443–53 (2004).
